# Supplementary material for: Condition-specific genetic interaction maps reveal crosstalk between the cAMP/PKA and the HOG MAPK pathways in the activation of the general stress response
Source: Mol Syst Biol. 2015 Oct 7;11(10):829. doi: 10.15252/msb.20156451 (PMC4631200; doi:10.15252/msb.20156451)
Supplement: Supplementary file 1 — Expanded View Figures PDF [file msb0011-0829-sd1.pdf]

Expanded View Figures

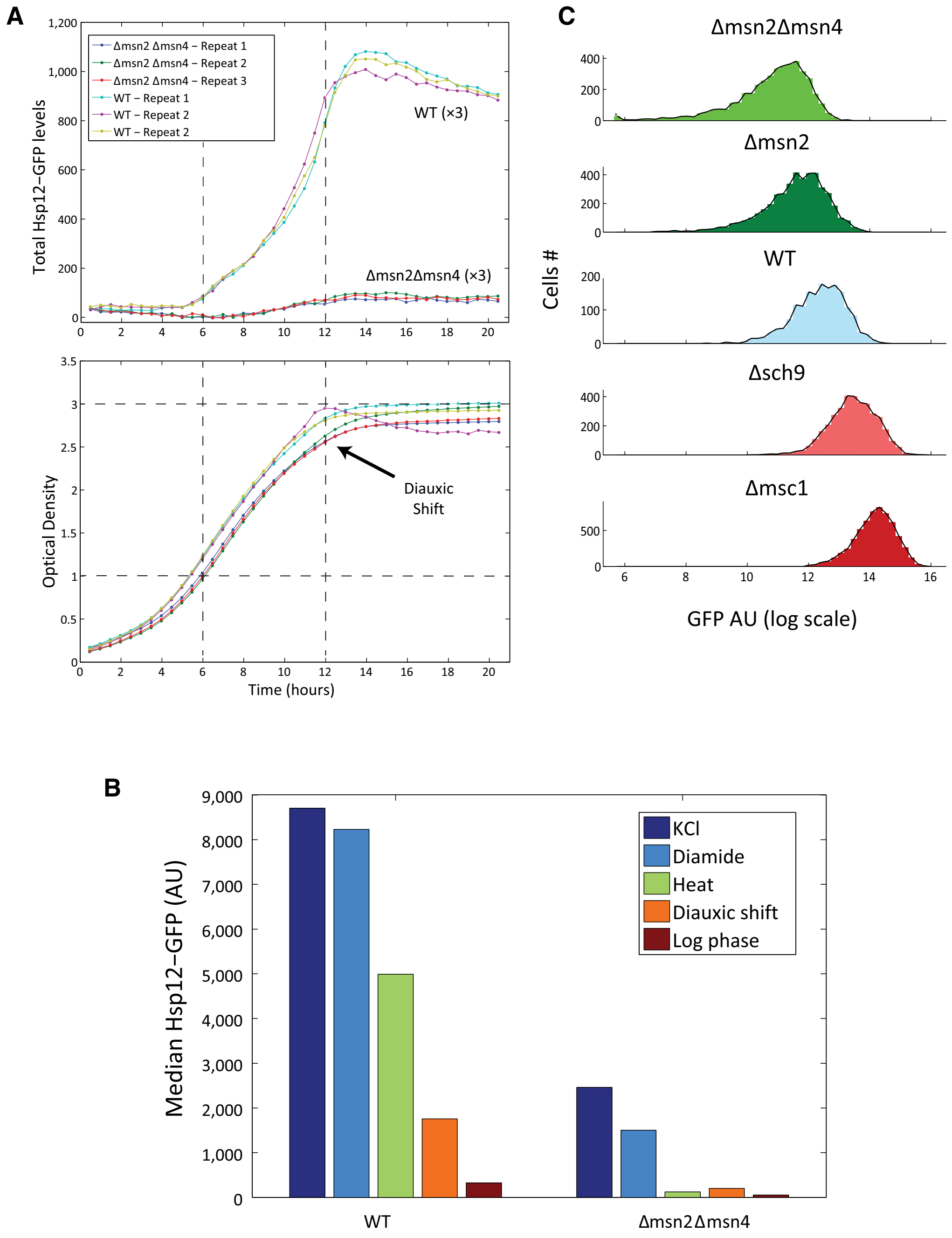

Figure EV1. Validity of reporter assay.

A *HSP12-GFP* is induced during late logarithmic growth phase. WT and Δ*msn2*Δ*msn4* strains carrying the *HSP12-GFP* reporter were grown overnight and diluted to OD ~0.2 at the beginning of the experiment. The strains were grown for 21 h in 96-well microtiter plate. The fluorescence (top panel) and optical density (OD, bottom panel) of each well were measured every ~30 min in a fluorescent plate reader (BioTek, Synergy S1).

B *HSP12-GFP* expression is mostly Msn2/4 dependent. Median Hsp12-GFP levels of WT and Δ*msn2*Δ*msn4* strains were measured using flow cytometry in different stress and growth conditions (Materials and Methods).

C Fluorescence levels of strains carrying *HSP12-GFP* reporter were measured using a flow cytometer, 90 min after the exposure to KCl stress. The histograms of the GFP levels in populations of different mutants are shown.

 © 2015 The Authors

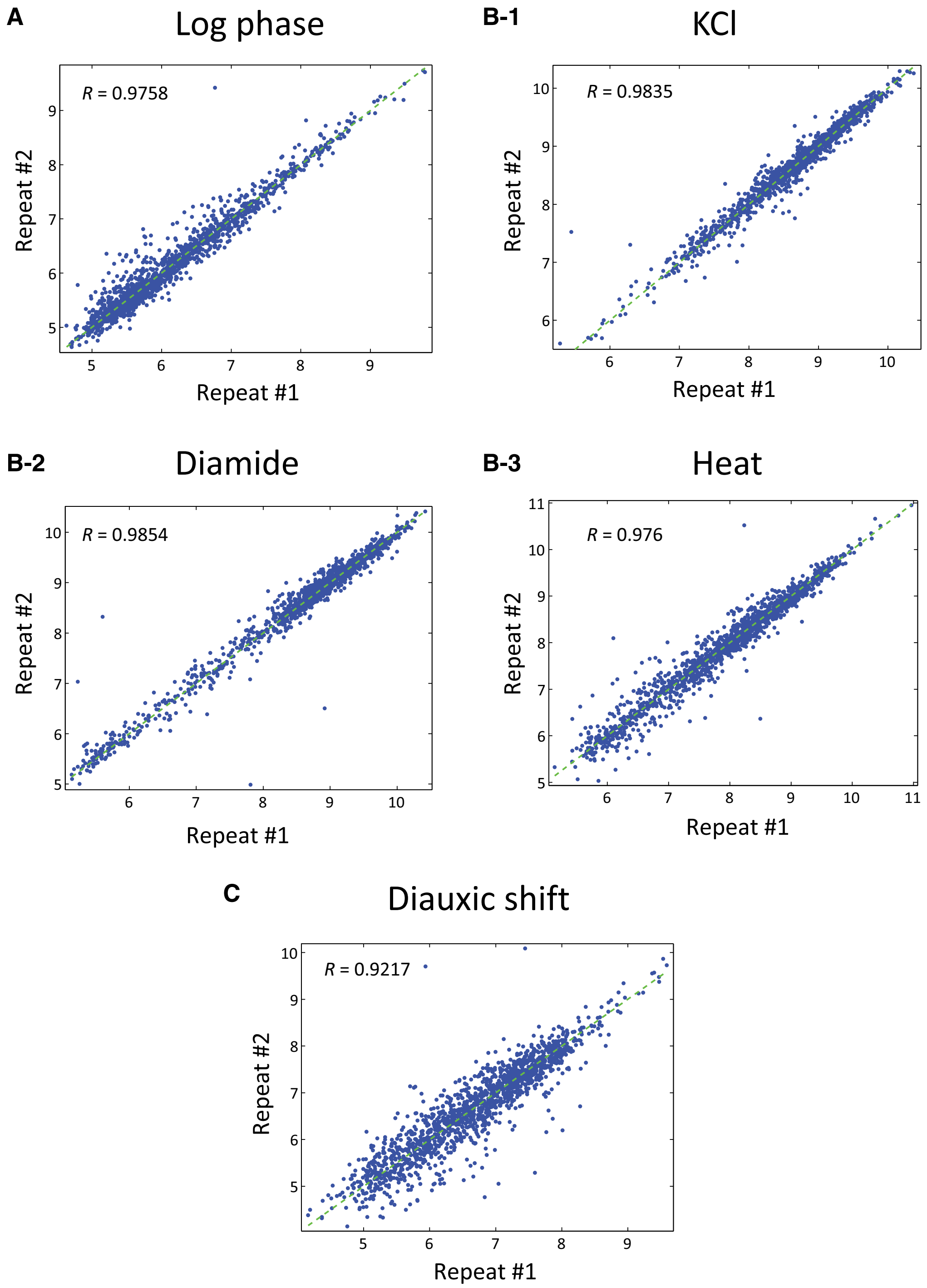

Figure EV2. Reproducibility of flow cytometry data.

A–C Comparison of two independent repeats of the different growth and stress conditions (Materials and Methods). Scatter plot of the median Hsp12-GFP levels (log scale) of 1,566 mutant strains in the two repeats.

© 2015 The Authors

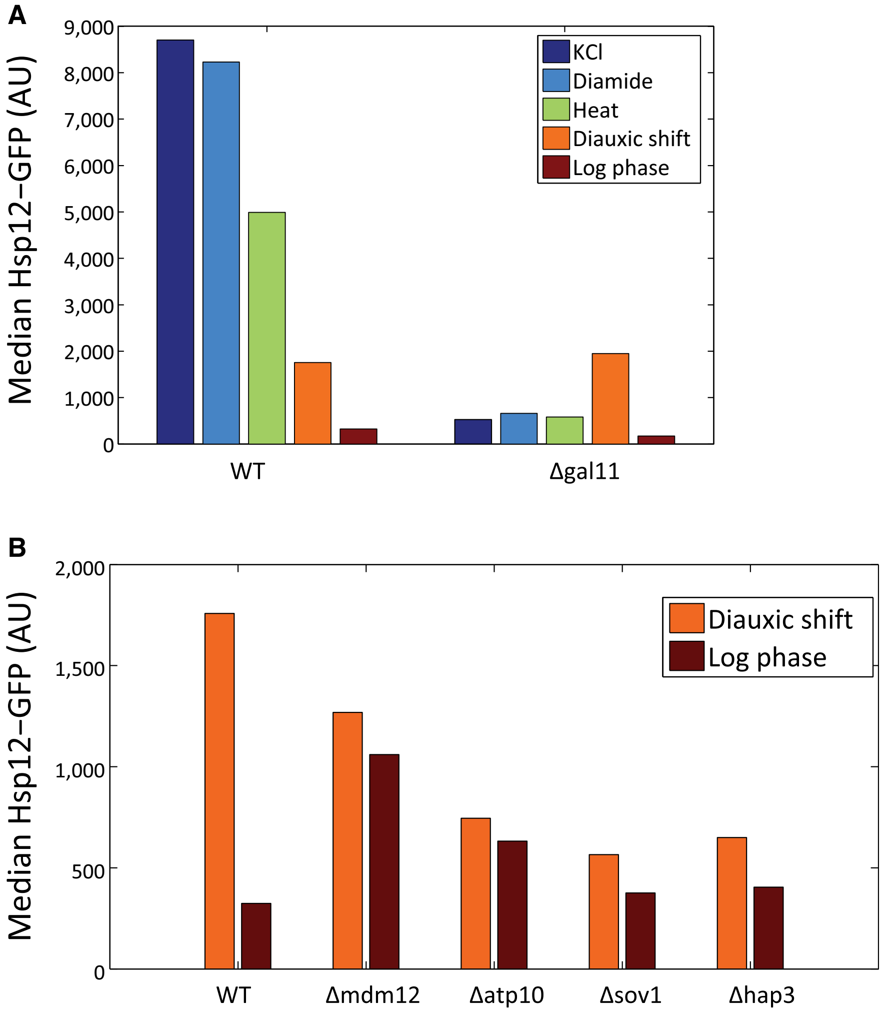

Figure EV3. Stress-specific behaviors.

A Hsp12-GFP levels of WT and Δ*gal11* strains in stress and growth conditions are compared. Although Δ*gal11* abolishes the expression of *HSP12-GFP* in stress conditions and in log phase growth, the levels of Hsp12-GFP in post-diauxic growth are higher than in WT.

B Hsp12-GFP levels of WT and strains with mutations in mitochondrial genes in two growth conditions are compared. Although the deletion of those genes reduced the levels of Hsp12-GFP in post-diauxic shift, the levels in log phase increased.

© 2015 The Authors

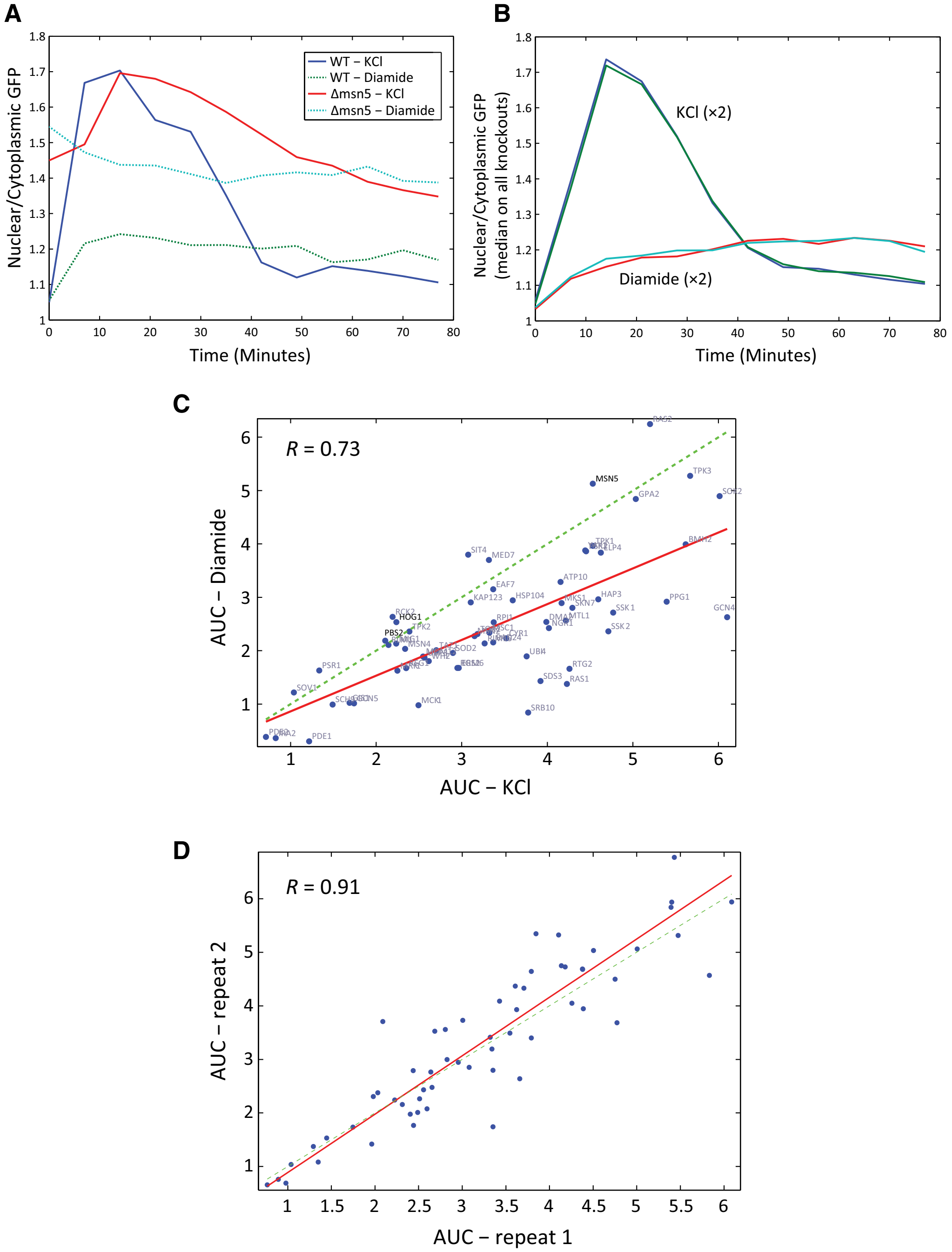

Figure EV4. Localization of Msn2-GFP.

A The ratio between the nuclear and the cytoplasmic Msn2-GFP over time after the exposure to stress is shown. In the Δ*msn5* strain, we observe high nuclear localization of Msn2-GFP at the beginning of the experiment, compared to WT.

B Msn2-GFP nuclear localization shows a stress-specific prototypical profile. The plot compares the dynamics of Msn2-GFP localization in KCl and diamide stress conditions. The average dynamics profile of all the examined strains in two independent repeats is shown.

C Msn2-GFP nuclear localization is correlated between stress conditions. The area under the curve (AUC) of Msn2-GFP localization dynamics was quantified for each mutant and stress condition (Materials and Methods). Scatter plot of the AUC values in KCl and diamide for all strains is shown.

D Two biological repeats of localization experiment lead to similar AUC values. Scatter plot of the AUC estimated for different strains after the exposure to KCl in two independent experiments.

© 2015 The Authors

A

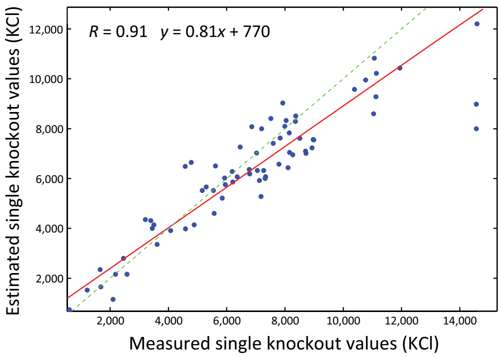

B

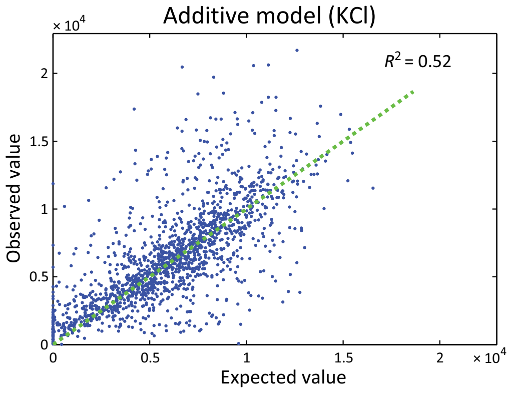

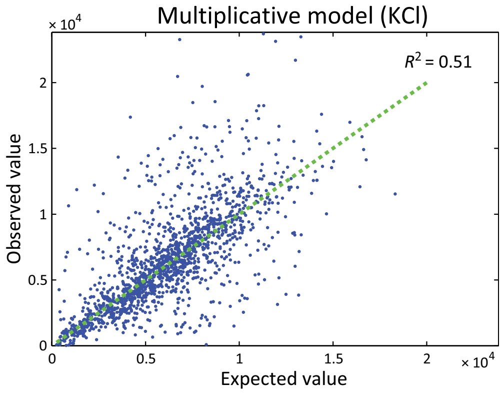

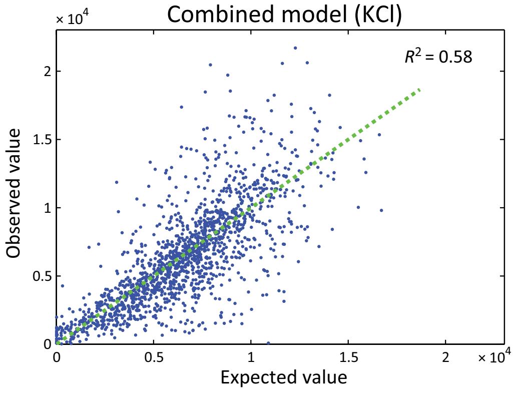

Percent of variance explained
(observed vs. expected)

	KCl	Diamide	Heat	Diauxic shift	Log phase
Additive	0.52	0.52	0.55	0.44	0.42
Multiplicative	0.51	0.50	0.54	0.41	0.33
Combined	0.58	0.59	0.64	0.55	0.64

C

	KCl	Diamide	Heat	Diauxic shift	Log phase
Additive vs. Msn dependent	0.22	0.47	0.22	0.16	0.80
Additive vs. Msn independent	0.57	0.26	0.57	0.51	0.22
Multiplicative vs. Msn dependent	0.51	0.33	0.78	0.78	−0.04
Multiplicative vs. Msn independent	0.06	0.23	−0.08	0.06	0.24

Figure EV5. Double-knockout library and genetic interactions.

A Median of double-knockout Hsp12-GFP values is a good estimator of single knockout values. Scatter plot of the median Hsp12-GFP levels after KCl stress of a knockout across all the knockouts it participates in (*y*-axis) versus the measured value of the single knockout (*x*-axis).

B Comparison between the observed Hsp12-GFP values to the expected values according to additive, multiplicative, and combined interaction models (Materials and Methods). The scatter plots show the comparisons in KCl stress. The percent of variance explained (R^2) using each model in other conditions is summarized in the table.

C The correlations coefficients (*R*) between the additive/multiplicative values of each knockout to its Msn2/4-dependent/independent effects (see scatters in Fig 4C).

 © 2015 The Authors

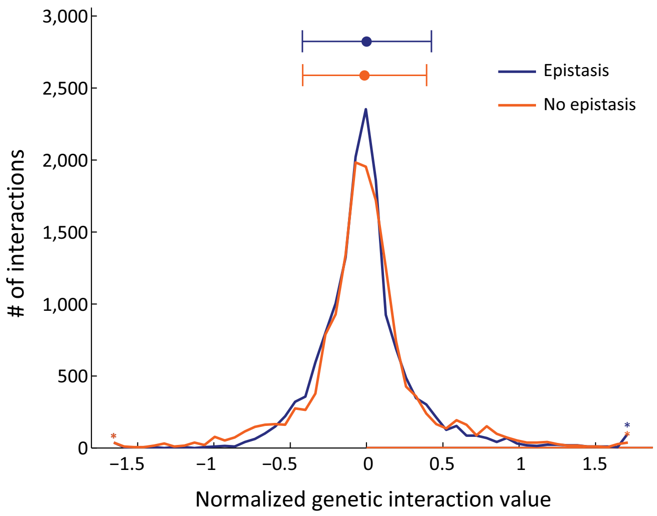

Figure EV6. Comparison between epistatic and non-epistatic genetic interactions.
The genetic interactions in stress and diauxic shift were divided into epistatic and non-epistatic (Materials and Methods) and the distribution of the values in each group is shown. The value of genetic interaction (Materials and Methods) in each condition was normalized according to the WT levels. Epistatic interactions tend to have either neutral or extreme interaction values.

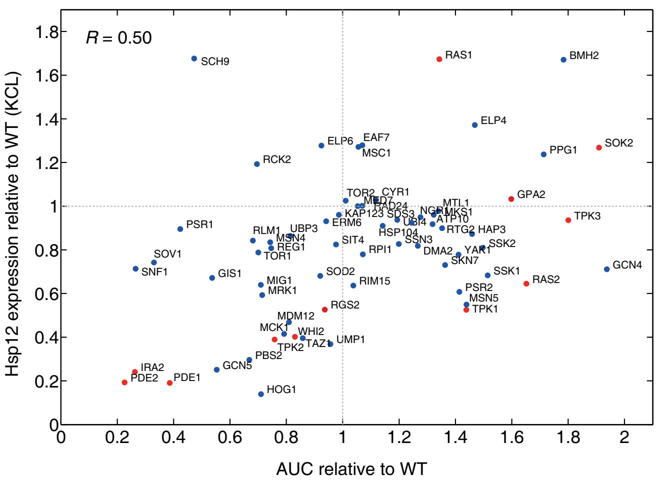

Figure EV7. Correlation between Hsp12-GFP levels and Msn2 localization.
The correlation between Hsp12-GFP levels upon osmotic stress and the nuclear accumulation of Msn2-GFP in the same condition in all the examined knockouts. Genes from the cAMP/PKA pathway (Fig 5C) are marked in red.

© 2015 The Authors
